# Folate Deficiency Triggers an Oxidative-Nitrosative Stress-Mediated Apoptotic Cell Death and Impedes Insulin Biosynthesis in RINm5F Pancreatic Islet β–Cells: Relevant to the Pathogenesis of Diabetes

**DOI:** 10.1371/journal.pone.0077931

**Published:** 2013-11-04

**Authors:** Hung-Chih Hsu, Jeng-Fong Chiou, Yu-Huei Wang, Chia-Hui Chen, Shin-Yi Mau, Chun-Te Ho, Pey-Jium Chang, Tsan-Zon Liu, Ching-Hsein Chen

**Affiliations:** 1 Graduate Institute of Clinical Medical Sciences, College of Medicine, Chang Gung University, Taoyuan, Taiwan; 2 Department of Physical Medicine and Rehabilitation, Chia-Yi Chang Gung Memorial Hospital, Chia-Yi, Taiwan; 3 Department of Nursing, Chang-Gung University of Science and Technology, Chia-Yi, Taiwan; 4 Cancer Center and Department of Radiation Oncology, Taipei Medical University and Hospital, Taipei, Taiwan; 5 Translational Research Laboratory, Cancer Center, Taipei Medical University and Hospital, Taipei, Taiwan; 6 Department of Microbiology, Immunology and Biopharmaceuticals, Collage of Life Sciences, National Chiayi University, Chiayi City, Taiwan; Broad Institute of Harvard and MIT, United States of America

## Abstract

It has been postulated that folic acid (folate) deficiency (FD) may be a risk factor for the pathogenesis of a variety of oxidative stress-triggered chronic degenerative diseases including diabetes, however, the direct evidence to lend support to this hypothesis is scanty. For this reason, we set out to study if FD can trigger the apoptotic events in an insulin-producing pancreatic RINm5F islet β cells. When these cells were cultivated under FD condition, a time-dependent growth impediment was observed and the demise of these cells was demonstrated to be apoptotic in nature proceeding through a mitochondria-dependent pathway. In addition to evoke oxidative stress, FD condition could also trigger nitrosative stress through a NF-κB-dependent iNOS-mediated overproduction of nitric oxide (NO). The latter compound could then trigger depletion of endoplasmic reticulum (ER) calcium (Ca^2+^) store leading to cytosolic Ca^2+^ overload and caused ER stress as evidence by the activation of CHOP expression. Furthermore, FD-induced apoptosis of RINm5F cells was found to be correlated with a time-dependent depletion of intracellular gluthathione (GSH) and a severe down-regulation of Bcl-2 expression. Along the same vein, we also demonstrated that FD could severely impede RINm5F cells to synthesize insulin and their abilities to secret insulin in response to glucose stimulation were appreciably hampered. Even more importantly, we found that folate replenishment could not restore the ability of RINm5F cells to resynthesize insulin. Taken together, our data provide strong evidence to support the hypothesis that FD is a legitimate risk factor for the pathogenesis of diabetes.

## Introduction

Diabetes is a complicated metabolic disorder which is characterized by a disturbance in the homeostasis between the control of glucose levels and insulin sensitivity. Diabetes has become an epidemic disease and remains a major public health issue owing to the fact that it poses a tremendous economic burden on individuals and health care system worldwide [1]. These emerging facts undersore the importance of identifying potential risk factors and understanding the mechanism(s) that lead to the disease. Information of this sort can be of value in the future development of intervention strategies against this chronic disease.

Oxidative stress is thought to be a major risk factor in the onset and progression of a variety of chronic degenerative diseases including diabetes [2], [3]. The role of oxidative stress in the insulin signaling process and a variety of risk factors that alter insulin sensitivity through mechanisms linked to oxidative stress have been postulated. Many of the common risk factors, such as obesity, increased age, unhealthy eating habit and a sedentary life style, all contribute to an oxidative environment that may alter insulin sensitivity either by increasing insulin resistance or impairing glucose tolerance. Despite the advances of these knowledges, the evidence linking a possible deficiency of a particular dietary micronutrient, such as folate, with the development of diabetes has thus far been scanty.

An adequate daily dietary intake of folate plays a pivotal role in maintaining a threshold blood level of this micronutrient in supporting several metabolic pathways, especially the methionine/homocysteine (Hcy) cycle [4]. It is well documented that the intracellular pool of folate is involved in the regulation of Hcy metabolism by supplying 5-methyltetrahydrofolate (5-methylTHF), which is necessary for the cellular methylation of Hcy back into methionine. Thus, the impairment of remethylation process due to the depletion of folate coenzymes will result in the accumulation of Hcy and enhanced production of reactive oxygen species (ROS), such as hydrogen peroxide (H_2_O_2_), and eventually lead to DNA hypomethylation [5]–[9]. Under this situation, the intracellular redox status can be shifted in favor of pro-oxidant state leading to oxidative stress environment. We have previonsly demonstrated that folate deficiency (FD) could trigger the downregulation of intracellular GSH and antioxidant enzymes, particularly H_2_O_2_-metabolizing catalase (CAT) and GSH peroxidase (GPx), and increased susceptibility of human hepatoma Hep G2 cells to various oxidant stress-induced cytotoxicity [10]. Thus, we hypothesize that FD could exacerbate the oxidative stress status in the insulin-producing pancreatic islets RNm5F cells because both intrinsic and extrinsic expressions of H_2_O_2_-metabolizing CAT and GPx in both tissues and cells have been reported to be extremely low [11]–[14]. Based on the above-noted rationale, we hypothesize that pancreatic β-cells, such as rat RINm5F cells, are likely to be especially vulnerable to FD-induced oxidative and nitrosative stress-mediated damages owing to their intrinsically low expression of hydrogen peroxide (H_2_O_2_)- inactivating enzymes, such as CAT and GPx. In addition, glutathione (GSH), the major thiol redox buffer, can provide a primary defense against oxidative stress by the ability to scavenge free radicals or participate in the reduction of H_2_O_2_ through its interaction in tandem with the enzymes GPx and GSSG reductase (GR) [15], [16]. Therefore, GSH may be especially important for β-cell antioxidant defense. Thus, the major focus of our current study is to obtain evidence to substantiate that FD is not only a risk factor for the progression of diabetes, but also a legitimate causative factor for this chronic degenerative disease.

## Materials and Methods

### Materials

Folate, amino acids, nucleosides, nucleotides and other chemical compounds were purchased from Sigma Chemical Co. (St. Louis, MO, USA). Minimal essential medium/alpha modified (αMEM) without ribosides, ribotides, deoxyriboside, deoxyribotides, glycine, serine, and folate was specially ordered and formulated by JHR (Lenex, KS, USA). Fetal bovine serum (FBS) was purchased from HyClone Laboratories (Logan, UT, USA). Penicillin, streptomycin, fungizone, and trypsin were ordered from GIBCO Laboratories (Grand Island, NY, USA).

### Cell Culture

RINm5F cells was obtained from the National Development Center of Biotechnology (Taipei, Taiwan). RINm5F cells were maintained as monolayer culture in complete medium at 37°C in a humidified 5% CO_2_ incubator with medium changed every two days. Complete medium contained αMEM with folate (2 µmole/L), thymidint (36 µmole/L), hypoxanthine (36 µmole/L), glycine (600 µmole/L), serine (250 µmole/L) and 10% fetal bovine serum. Penicillin (20,000 units/L), streptomycin (20 mg/L), and fungizone (2.5 mg/L) are also added to media for elimination of contamination. To formulate folate-deficient media, folate as well as thymidine, hypoxanthine, and glycine were omitted from complete media to stress substrate availability in one carbon metabolism. To minimize exogenous folate source, fetal bovine serum was replaced with dialyzed fetal bovine serum (dFBS), which has been dialyzed at 4°C for 16 hours against 6×10 volumes of sterile PBS. Control medium was complete medium with 10% dFBS. Therefore, RINm5F cells cultured in folate deficient medium (in the absence of folate and thymidine, hypoxanthine, glycine and serum) are designated as folate-deficient cells (FD). RINm5F cells cultured in the control medium are referred to as control cells (FC).

### Measurement of Intracellular Reactive Oxygen Species (ROS)

The production of intracellular ROS was detected by flowcytometry using DCF-DA probe. RINm5F cells were grown under FC or FD condition for various time periods until 80% confluency was reached. The culture medium was then replaced with new medium and treated with 10 µM DCF-DA for 30 min in the dark, washed once with PBS, detached by trysinization, collected by centrifugation, and resuspended in PBS. The intracellular ROS, as exhibited by the fluorescence of DCF, was measured with a Becton-Dickinson FACS-Calibur flow cytometer. Direct measurement of hydrogen peroxide (H_2_O_2_) in the medium cultivated under FC or FD condition for RINm5F cells was determined by the Amplex red H_2_O_2_/peroxidase assay kit (Invitrogen, Molecular probes) according to the instruction of the manufacturer. Measurement of hydroxyl radical (OH^•^) formation was performed flowcytometrically using hydroxyphonyl fluorescein (HPF) as a probe. [17], [18].

### Determination of Homocysteine Concentration

After RINm5F cells cultivated under FC or FD condition for a specified time period, they were seeded in 24-well microculture plates and allowed to adhere overnight. The cells were then washed once with PBS (pH7.4) followed by adding 2.0 ml of the fresh medium and cultured for 72-hr. The homocysteine levels of the culture medium were then analyzed by an automated autoanalyzer [Hitachi 917 (7170)] based on the total homocysteine biochemical assay kit developed by Formosa Biomedical Technology Co. The principle of the assay is based on the cleavage of homocysteine by a genetically engineered homocysteine α,γ-lyase to produce hydrogen sulfide (H_2_S) which can be determined spectrophotometrically.

### Detection of Intracellular Nitric Oxide (NO) Production

Intracellular NO was detected by confocal microscopy using DAF-FM probe. RINm5F cells grown under FC or FD condition for various time periods were treated with 10 µM DAF-FM for 20 min at 37°C in the dark, then washed twice with PBS buffer. The DAF-FM fluorescence reflecting the level of intracellular NO was measured by a Leica confocal microscopy with excitation wavelength set at 488 nm and emission wavelength at 520 nm. For confirmation of FD-induced NO production, we used the Griess diazotization kit purchased commercially (Molecular Probes).

### Determination of Total Intracellular Glutathione

Glutathione (GSH) of various cell supernatants were assayed simultaneously using the method of Hilf et al. [19]. Briefly, GSH was determined using 0.5 ml of cell supernatant. To this sample, 4.5 ml of the phosphate-EDTA buffer (pH 8.0) was added. The final assay mixture (2.0 ml) contained 100 µL of the diluted supernatant, 1.8 mL of phosphate-EDTA buffer, and 100 µL of the o-phthalaldehyde (OPT) solution (100 µg). After through mixing and incubation at room temperature for 15 min, the solution was transferred to a quartz cuvette. Fluorescence at 420 nm was determined following activation at 350 nm. FD-mediated intracellular GSH depletion was further confirmed by confocal microscopic observation using CMF-DA staining technique. CMF-DA, a nonfluorescent compound, can enter the intracellular compartment and reacts with esterase to allow CMF trapped inside the cell. The interaction of CMF with thiol group of GSH will result in the generation of green fluorescence. Thus, the decrease in fluorescence reflects the depletion of the availability of GSH. In this experiment, RINm5F cells grown under FC and FD conditions were loaded with 10 µM CMF-DA for 30 min at 37°C and the levels of intracellular GSH, as reflected by multiphoton fluorescence images were then obtained by a Leica SP2MP laser scanning confocal microscpy (Leica-Microsystems, Wetzlar, Germany).

### Measurement of △Ψm by flowcytometry

Rhodamine 123 is a fluorescent dye that is incorporated into mitochondria in a △Ψm-dependent manner. After RINm5F cells grown under FC or FD condition for a designated period, the culture medium was replaced with a new medium containing 5 µM rhodamine 123 for 30 min in the dark. Subsequent to the incubation step, cells were harvested by trypsinization, following which, △Ψm, as indicated by the fluorescence level of rhodamine 123, was analyzed using a Becton-Dickinson FACS-Calibur flow cytometer.

### Detection of Cytosolic Calcium Level

Cytosolic calcium level was visualized by confocal microscopy using Fluo-4 probe. RINm5F cells were cultivated under FC or FD condition for 2-week. At the end of the cultivation period, the medium was replaced with new medium and treated with 2 µM Fluo-4 for 30 min at 37°C in the dark. After loading, both types of cells were rinsed 3 times with HEPES-buffered saline. Fluo-4 fluorescence reflecting the level of cytosolic calcium was visualized by a Leica confocal microscopy.

### MTT Test

Cells were seeded in 96 wells plates with a density of 1×10^4^ cells/well. After 24 hours, the media were replaced with 10 µL MTT [3-(4,5-dimethylthiazol-2- yl)-2.5-diphenyl tetrazolium bromide] (1 mg/mL). The plates were allowed to stand for 4 hrs before they were added 100 µL of 15% sodium dodecyl sulfate for 24 hr to thoroughly dissolve the dark blue crystals. Cell viability was determined by absorbance at 570 nm measured by an ELISA reader.

### Tunel Assay

Apoptotic cell death was assayed by using an Apo-BrdU in situ DNA fragmentation assay kit (Promega, Medison, WI, USA). This kit measures the fragmented DNA of apoptotic cells by catalytically incorporating fluorescein-12- dUTP at 3′-OH DNA ends using the terminal deoxynucleotidyl transferase enzyme. The fluorescein-12-dUTP-labeled DNA can then be quantitiated by flowcytometry. In this study, RINm5F cells were cultivated in either FC or FD condition for two weeks and the fluoressein-12-dUTP-labeled fragmented DNA of apoptotic cells were quantified using Becton-Dickinson FACS-Calibur flow cytometer.

### DNA Fragmentation Assay

After RINm5F cells grown under FC and FD conditions for two weeks, they were lysed in a buffer containing 10 mM Tris (pH 7.4), 150 mM NaCl, 5 mM EDTA and 0.5% Triton X-100 for 30 min on ice. Lysates were vortexed and cleared by centrifugation at 10,000 g for 20 min. Fragmented DNA in the supernatant was extracted with an equal volume of neutral phenol : chloroform : isoamylalcohol mixture (25∶ 24∶ 1) and analyzed electrophoretically on 2% agarose gel containing 0.1 µg/ml of ethidium bromide.

### Wertern Blot Analysis

Cells plated at 1×10^6^ per 10-cm culture dishes were allowed to grow for 24 hrs at 37°C in a 5% CO_2_ incubator. Protein samples were collected in RIPA buffer (50 mM Tris • HCl, pH 7.5, 150 mM NaCl, 1 mM EDTA, 1% Triton X-100, 0.25% sodium deoxycholate, 0.1% SDS) containing 50 mM sodium fluoride, 1 mM sodium orthovanadate, 10 mM β-glycerophosphate and 1× protease inhibitor cocktail (Sigma-Aldrich, St Louis, MO). Protein concentrations were assayed using Bio-Rad protein assay kit (Bio-Rad. Richmond, CA). Protein extracts representing 40 µg were separated on a 12% SDS-PAGE gel and electrophoretically transferred to a polyvinylidine difluoride (PVDF) membrane. Membranes were blocked in 5% skim milk powder in TBST solution (Tris-buffered saline containing 0.1% Tween20) for 1 hr at room temperature followed by incubation with first antibodies against. β-catenin (0.3 µg/ml) (Upstate), iNOS (1∶ 250) (BD Bioscience), Bcl-2 (1∶ 1000), p65 (1∶10000) (Santa Cruz Biotech), Bax (Chemico Int.) (Temecula, CA), CHOP (1∶1000) and actin (1∶ 10,000) (Sigma-Aldrich. St. Louis, MO). Following washing with TBST solution, blots were incubated with the appropriate HRP-labeled secondary antibody for 1 hr at room temperature. The antigen-antibody complexes were detected by the enhanced chemiluminescence (ECL) with a chemiluminescence analyzer.

### Quantification and Immunofluorescent Detection of Insulin

RINm5F cells were cultivated in either FC or FD condition for one to two weeks. After that, RINm5F cells were analyzed and visualized for the distribution of insulin by confocal microscope. Insulin was detected with fluorescein (FITC)-conjugated goat anti-rabbit antibody (green), and nuclear counterstaining was performed with DAPI (blue). In addition, we also quantified insulin levels for RINm5F cells cultivated under FC and FD condition for one to two-week period by use of a one-step immunoassay kit purchased commercially (ARCHITECT insulin assay, Abbott Laboratory) based on a chemiluminescent microparticle immunoassay (CMIA) principle. Furthermore, we also performed an experiment on insulin secretion in response to stimulation by different glucose concentrations (1.0 to 3.0 g/L, respectively) in FC and FD cells.

### Statistical Analysis

Data are presented as mean ± SD or mean ± SE. Comparison of two groups was made using Student’s test. An associated p value of <0.05 was considered significant.

## Results

### FD Triggered Oxidative-nitrosative Stress in RINm5F Cells

**Figure 1:**
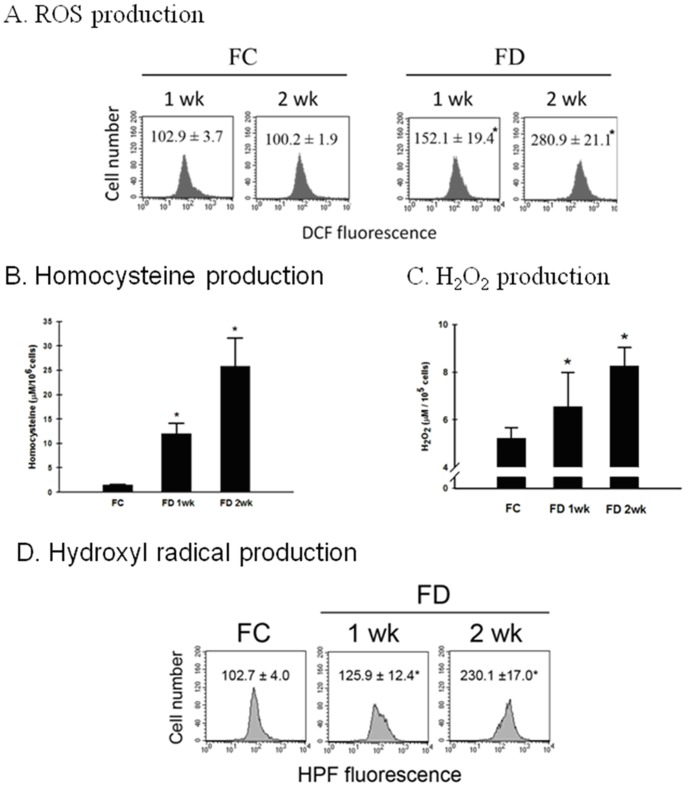
Folate deficiency triggers oxidative stress in RINm5F cells. (A) Production of intracellular ROS in the designated cells cultivated under either FC or FD condition was evaluated flowcytometrically using DCF-DA as the probe. At the end of 2-week cultivation period under FD condition, a nearly 3-fold increase in ROS production, as reflected by the DCF fluorescence intensity, could be detected as compared to FC control. The values shown are mean ± standard errors (n = 3 of individual experiments). *p<0.05 vs. control. (B) The production of homocysteine (Hcy) was followed using an enzyme-assisted spectrophotometric detection of H_2_S production which is directly proportional to the concentration of Hcy. The results were expressed as µM Hcy/10^6^ cells. Each bar represents an average of triplicate determinations. *p<0.05 v.s control. (C) Direct measurement of H_2_O_2_ released by RINm5F cells using Amplex red hydrogen peroxide assay kit. The results were expressed as µM H_2_O_2_/10^5^ cells. It is apparent that H_2_O_2_ production by RINm5F cells cultivated under FD condition was higher than FC control. *p<0.05 v.s control. (D) The production of OH^•^ in either FC or FD condition of RINm5F cells was determined flowcytometrically using hydroxyphenyl fluorescein (HPF) as a probe. *p<0.05 v.s control.

**Figure 2:**
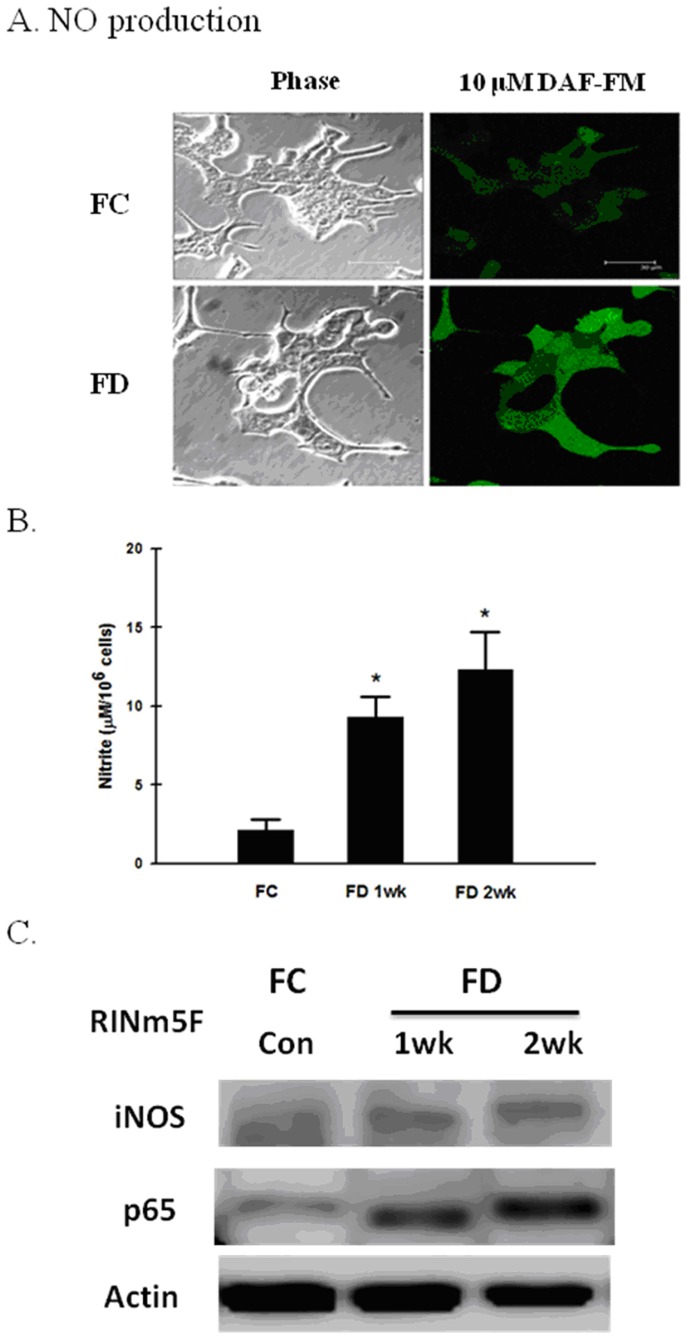
Folate deficiency provokes nitrosative stress by NF-κB-dependent iNOS activation mechanism independent of Hcy accumulation. (A) Production of nitric oxide (NO) was evaluated using probe-based confocal microscopic imaging technique. The overproduction of NO could be visualized in cells cultivated under FD condition. (B) NO production in FD RINm5F cells was confirmed using Griess reagent kit for nitrite determination. As expected, FD condition could indeed generate plethoric amounts of nitrite, an index of NO production. (C) FD-triggered overproduction of NO was linked mechanistically to NF-κB-dependent activation of iNOS.

The production of intracellular ROS in RINm5F cells cultivated under either FC or FD condition was evaluated flowcytometrically using the DCF-DA staining technique. A nearly 3-fold increase in DCF fluorescence intensity could be detected following RINm5F cells that were cultivated for 2-week period under FD condition as compared to FC counterparts (Fig. 1A). As also revealed in Fig. 1B, when RINm5F cells were grown under FC condition, the generation of homocysteine (Hcy) in the culture medium remains steadily low. However, the concentrations of Hcy rose sharply when cells were cultivated under FD condition. Furthermore, FD-induced increase in Hcy levels was found to be correlated with increased production of H_2_O_2_ (Fig. 1C). These data clearly demonstrated that FD-triggered oxidative stress was intimately associated with Hcy-dependent H_2_O_2_ production. Along this same vein, we also measured the production of OH^•^ in RINm5F cells cultivated under either FC or FD condition using hydroxyphenyl fluorescein (HPF)-based flowcytometric method. As expected, a more than 2-fold increase in OH^•^ production could be detected when RINm5F cells were grown under FD condition for 2-week as compared to the FC controls (Fig. 1D). As to the production of nitric oxide (NO) instigated by FD, we first evaluated this cellular event by the confocal microscopic imaging technique using NO-specific DAF-FM probe. As shown in Fig. 2A, overproduction of NO could be vividly seen in RINm5F cells cultivated for 2-week under FD condition as evident by strong green fluorescent intensity exhibited when compared to FC control. To confirm this observed phenomenon, we quantified the nitrite production, a spontaneous oxidation product of NO by the use of the Griess diazotization method (Fig. 2B). Mechanistically, we demonstrated that FD-triggered overproduction of NO was mediated via upregulation of inducible nitric oxide synthase (iNOS) through increased activation of NF-κB (Fig. 1C). These data clearly demonstrated that FD-induced nitrosative stress had no bearing with overproduction of Hcy as analogous to the finding reported in the literature [20].

### FD Triggered Intracellular GSH Depletion in RINm5F Cells

**Figure 3:**
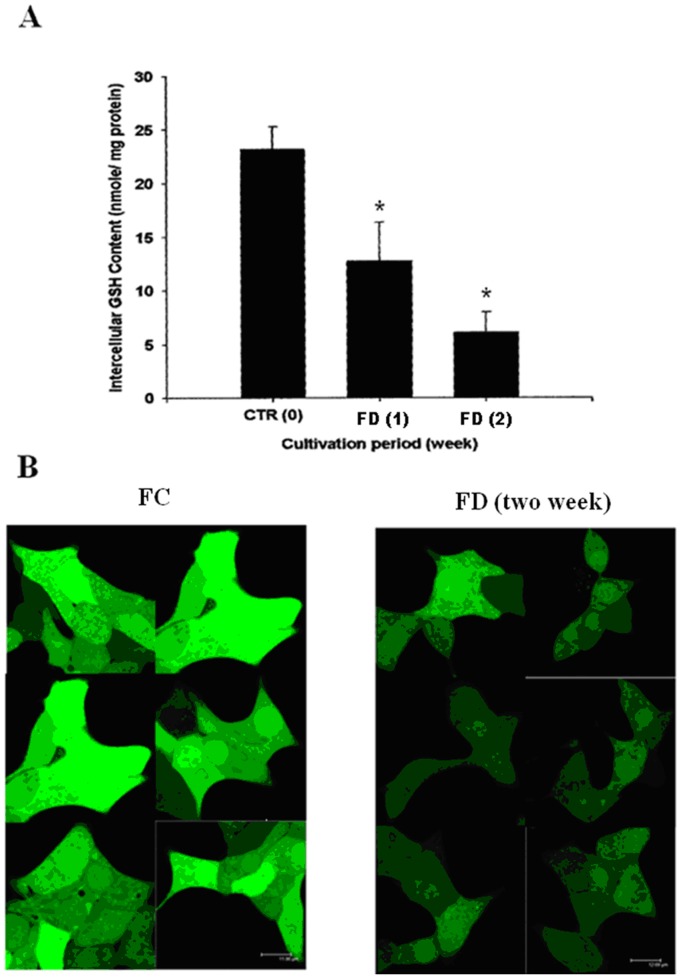
Folate deficiency triggers intracellular GSH depletion in RINm5F cells. (A) The fluorometric o-pathalaldehyde (OPT) method was used to quantify intracellular GSH contents in RINm5F cells cultivated under either FC or FD condition. At the end of cultivating cells under FD condition for 2-week period, total GSH contents were found to be depleted 75% as compared to their FC counterparts. Data shown are mean ± standard deviation of triplicate determinations. *p<0.05 vs. control. (B) Probe-based confocal microscopic imaging technique was used to confirm GSH depletion phenomenon instigated by FD. Under similar cultivation period (2-week), a drastic loss of the green fluorescence of cellular CMF-GSH conjugate associated with folate-deprived cells was a direct testimony of intracelluar GSH depletion.

We hypothesized that FD-induced oxidative-nitrosative stress could trigger intracellular GSH depletion. To test this possibility, we firstly quantified the total intracellular GSH contents of RINm5F cells cultivated under either FC or FD condition by an established fluorometric OPT method. In this study, we found that FD could induce a depletion of nearly 75% of total intracellular GSH contents when RINm5F cells were grown under FD condition for 2-week period. (Fig. 3A).

To further confirm this observed phenomenon, we used probe-based confocal microscopic imaging technique. After the probe (CMF-DA) entered into the intracellular compartment and cleaved by esterase, the trapped CMF moiety interacted with thiol group of GSH resulting in the generation of green fluorescence. The extents of GSH depletion could then be visualized based on the disappearance of green fluorescence. As shown in Fig. 3B, a drastic loss of the green fluorescence of CMF-GSH conjugate in RINm5F cells cultivated under FD condition for 2-week period is a direct testimony of intracellular GSH depletion.

### FD Evoked Cytosolic Calcium Overload and Induced a Drop of △Ψm in RINm5F Cells

**Figure 4:**
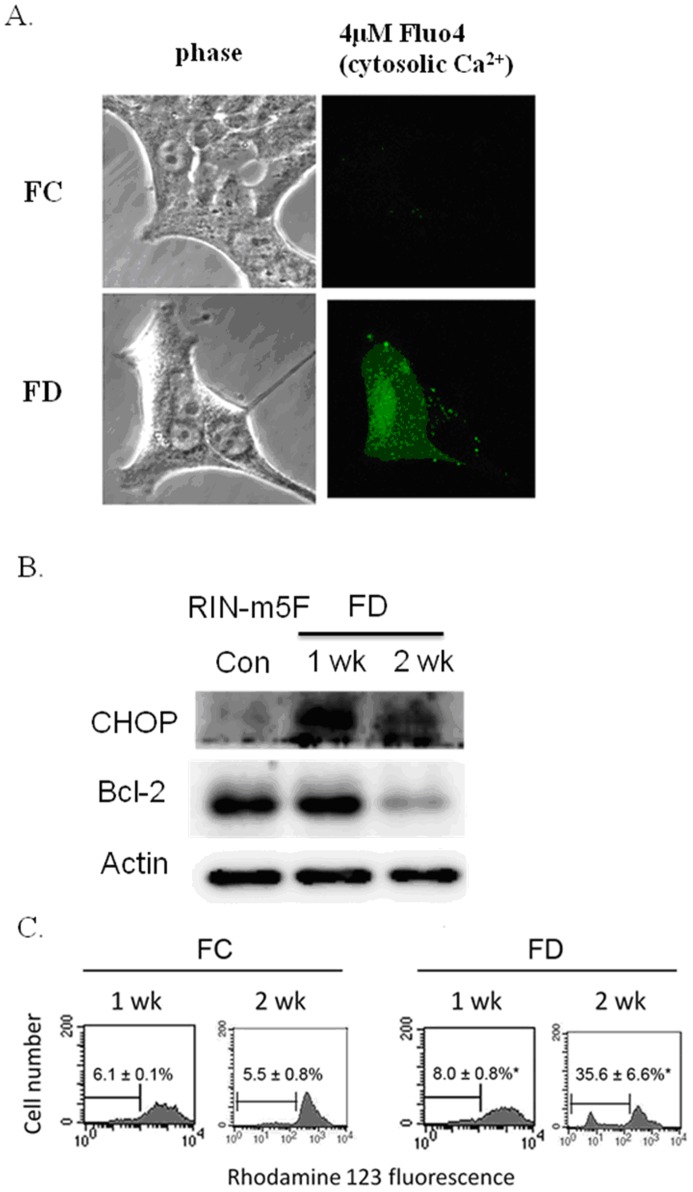
Folate deficiency induces cytosolic calcium (Ca^2+^) overload and causes disruption of △Ψm in RINm5F cells. (A) Probe-based confocal microscopic imaging technique was used to detect cytosolic Ca^2+^ overload. Under FC condition, cytosolic Ca^2+^, as reflected by the green fluorescence of Fluo-4 probe, was essentially non-detectable. Conversely, as a result of cultivating cells under FD condition for 2-week period, a cytosolic Ca^2+^ overload phenomenon could be detected. This phenomenon may be attributed to the depletion of ER Ca^2+^ by NF-κB-dependent iNOS-mediated overproduction of NO. (B) The interplay of NO and cytosolic Ca^2+^ provoked ER stress as evident by the activation of CHOP expression in RINm5F cells cultivated under FD condition. (C) △Ψm was evaluated by using the rhodamine 123 fluorescent dye and meansured flowcytometrically. The disruption of △Ψm, as reflected by the percentage of cells with depolarized mitochondrial membrane, was much higher in FD cells (35.6±6.6%) when compared to FC cells (5.5±0.8%). Apparently, FD could sensitize cells to undergo apoptosis. Values shown are mean ± standard errors of 3 independent experiments. *p<0.05 vs. control.

It has been established that ER Ca^2+^ stores are a target of NO. Excessive NO can deplete ER Ca^2+^ leading to ER stress and eventual apoptosis [21]. In this study, we evaluated cytosilic Ca^2+^ levels by confocal microscopic imaging technique using Fluo-4 as the probe. As shown in Fig. 4A, cytosolic Ca^2+^ level, as reflected by the green fluorescence of Fluo-4 probe, was essentially non-detectable in cells that had been cultivated under FC condition. Conversely, we found that cells grown under FD condition could induce cytosolic Ca^2+^ overload that was probably discharged from ER due to excessive NO generated. The interplay between excessive NO production and cytosolic Ca^2+^ deregulation was found to evoke ER stress as evident by the activation of CHOP expression (Fig. 4B).

An irreversible loss of mitochondrial membrane potential (△Ψm) has been established as a prerequisite process preceding apoptosis [22]. Thus, we proceeded to examine if RINm5F cells cultivated under FD condition could provoke a disruption of △Ψm. In this study, △Ψm was evaluated by using the rhodamine 123 fluorescent dye, which could specifically accumulate within the mitochondrial compartment in △Ψm dependent manner. As shown in Fig. 4C, disruption of △Ψm, as reflected by the percentage of cells with depolarized mitochondrial membrane, reached 35.6±6.6% when cells were cultivated under FD condition for 2-week which was considerably higher than FC control (5.5±0.8%).

### FD-induced Cell Death of RINm5F Cells was Apoptotic in Nature

**Figure 5:**
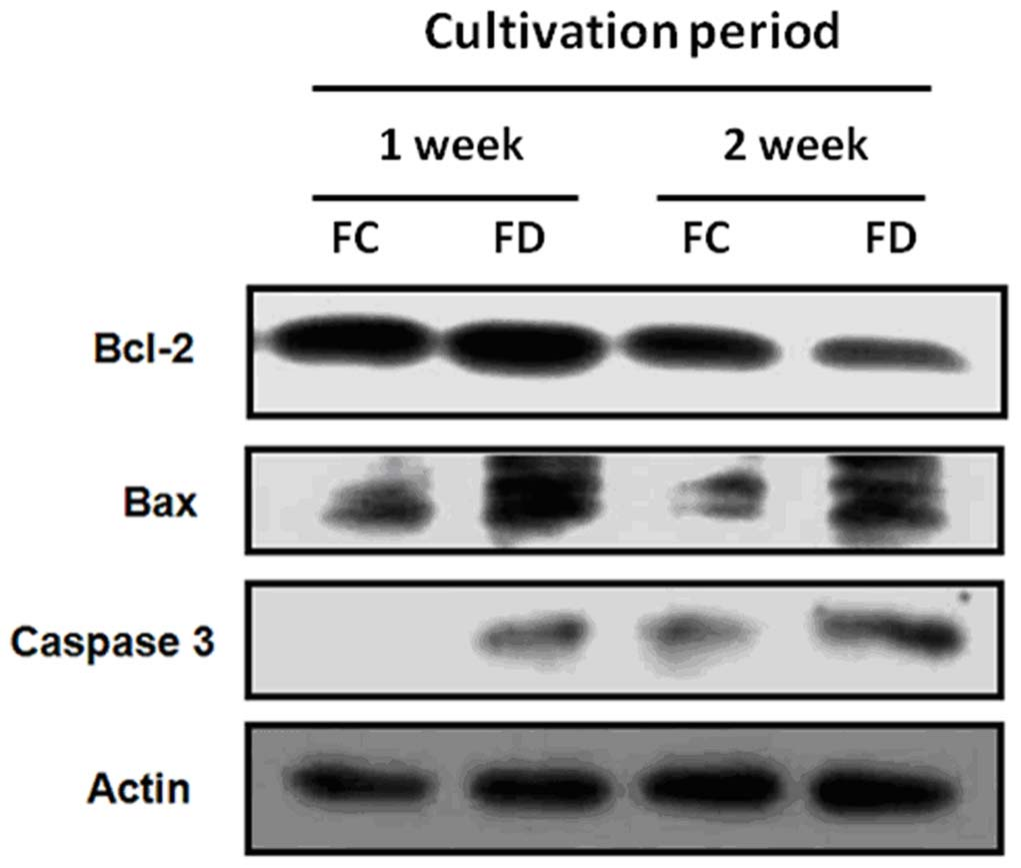
Folate deficiency-induced cell death of RINm5F cells is apoptotic in nature. At the end of cultivating cells under FD condition for 2-week period, the expression of proapoptotic Bax was augmented in tanderm with the downregulation of Bcl-2. Thus, FD condition could increase Bax/Bcl-2 ratio, a condition favoring apoptosis to occur. Concomitantly, an increase of Bax/Bcl-2 ratio is accompanied by the robust activation of caspase 3 activity.

**Figure 6:**
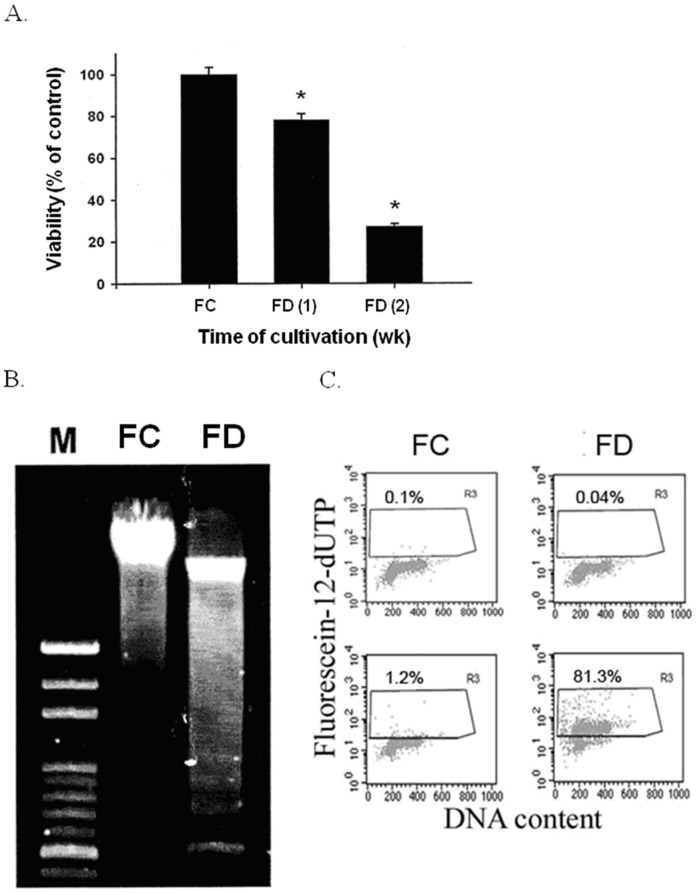
Folate deficiency-induced cell death and apoptosis was shown to be characteristically associated with DNA fragmentation, as demonstrated by either by the nucleosomal DNA fragmentation of 180–200 base pair multimers performed by gel electophoresis (B) or by drastic increase in the terminal transferase-mediated fluorescein-12-dUTP-labeled fragmented DNA (TUNEL-positive) of apoptotic cells detected flowcytometrically (C).

It has been documented that the Bcl-2 family significantly regulates apoptosis, either as an activator (Bax) or as an inhibitor (Bcl-2). Thus the Bax/Bcl-2 ratio is recognized as a key factor in regulating apoptosis process. Bax can dimerize with Bcl-2 and inhibits its function and thereby promotes apoptosis [23], [24]. In the current study, we demonstrated that cultivating RINm5F cells under FD condition for 2-week period could drastically increase in Bax/Bcl-2 ratio that fostering apoptosis by caspase-3 activation (Fig. 5). Consequently, FD-induced apoptotic cell death were shown to be characteristically associated with nucleosomal DNA fragmentation of 180–200 base pair multimers, and drastic increase in the terminal transferase-mediated fluorescein- 12-dUTP-labeled fragmented DNA (TUNEL-positive) of apoptotic cells (Figs. 6B and 6C).

### FD Impeded The Ability of RINm5F Cells to Synthesize and Secretion of Insulin

**Figure 7:**
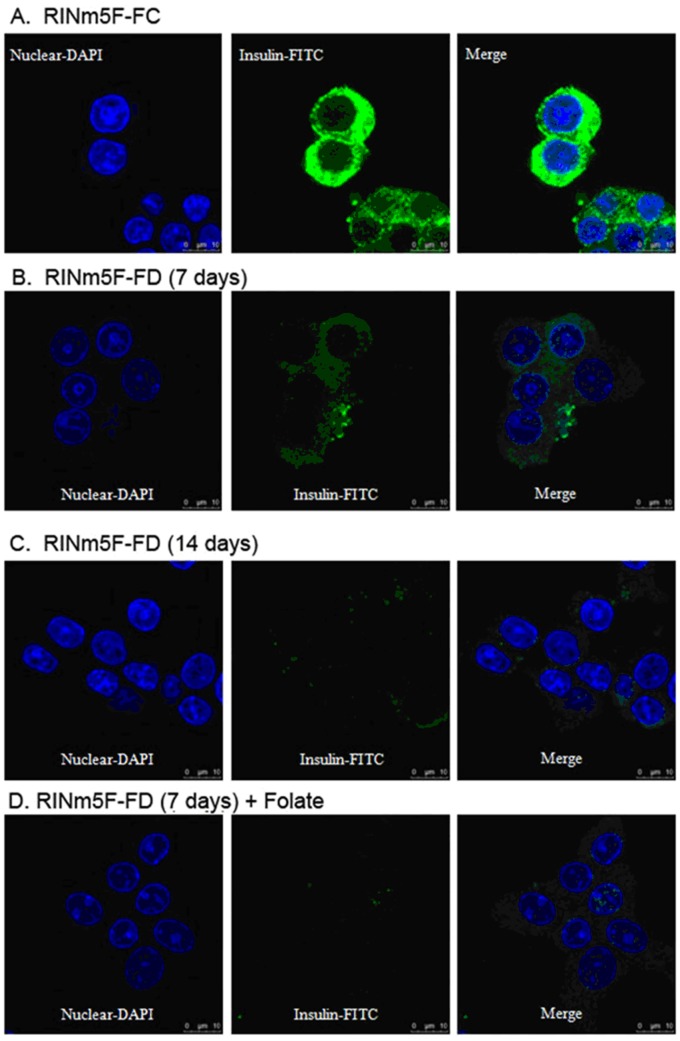
Folate deficiency impedes the ability of RINm5F cells to synthesize insulin. We evaluate the ability of RINm5F cells to synthesize insulin by immunocytochemical staining method and visualized by the confocal microscopic imaging process. FC cells were shown to be capable of synthesizing planteous amounts of insulin as reflected by strong green insulin-FITC fluorescence localized around the perinuclear region (A). Cells cultivating under FD condition for merely one-week period, the ability of cells to synthesize insulin were found to be severely lost (B). Even more impressively, cells suffered from folate deprivation for 2-week period, a nearly complete loss of the ability to synthesize insulin could be visualized (C). This process was shown to be irreversible since folate replenishment could not restitute the injurious cells to resynthesize insulin (D).

**Figure 8:**
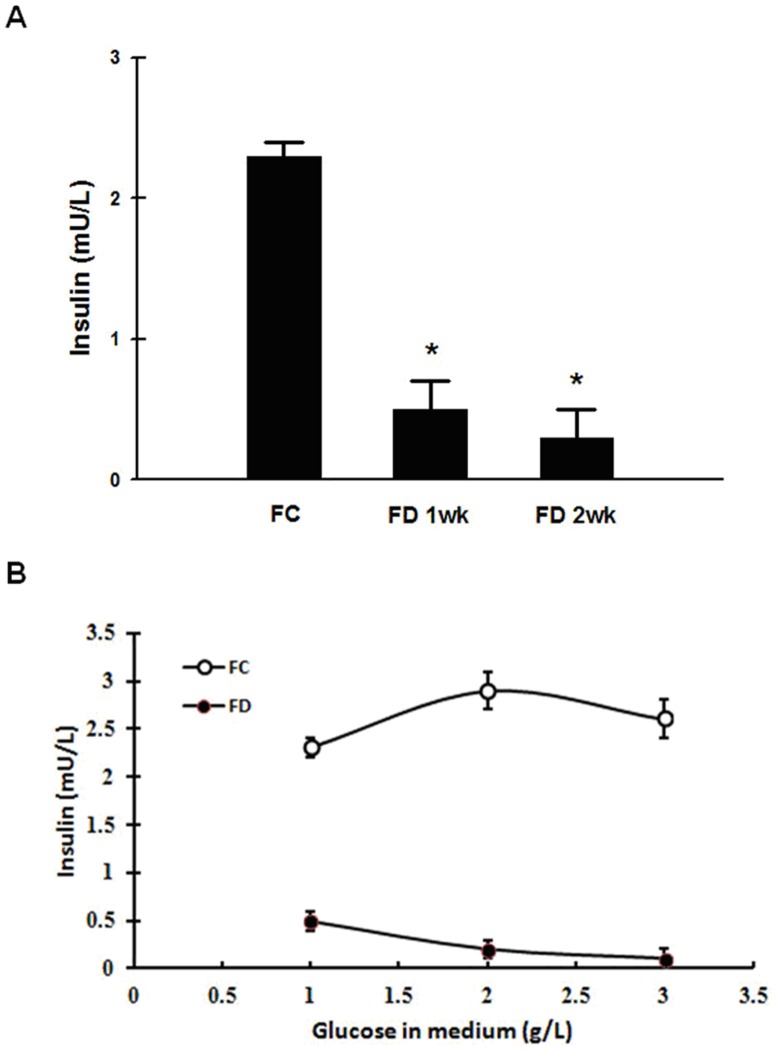
Folate deficiency simultaneously induces an impediment of insulin- synthesizing ability and insulin secretion in response to glucose challenge in RINm5F cells. (A) Direct quantification of insulin by a chemiluminesent microparticle immunoassay (CMIA) kit revealed that FD condition could hamper the ability of these cells to synthesize insulin. (B) FD condition could render RINm5F cells to loss their abilities to secrete insulin in response to glucose challenge. The values shown are mean ± standard deviation (n = 3 of individual experiments). *p<0.05 v.s. control.

The synthesis of insulin by RINm5F cells cultivated under either FC or FD condition was compared using immunocytochemical staining method and visualized by the confocal microscopic imaging process. As shown in Fig. 7, cells cultivated under FC condition had been demonstrated to be capable of synthesizing plenteous amounts of insulin as reflected by the strong insulin-FITC green fluorescence exhibited around the perinuclear region. Conversely, cells grown under FD condition for merely one-week, the green fluorescence of insulin-FITC was now found to be severely suppressed. At the end of cultivating cells for 2-week period under FD condition, a nearly complete abolishment of green fluorescence of insulin-FITC had ensued. These data imply that FD condition could dramatically hamper the ability of RINm5F cells to synthesize insulin (Fig. 7C). More importantly, we also demonstrated that replenishment of folate after a short-term deprivation of folate (e.g., one-week) could no longer be able to revert the ability of these cells to resynthesize insulin (Fig. 7D). This finding indicated that the injury imposed by FD toward RINm5F cells was non-revertible. To reconfirm the findings of confocal images, we also quantified insulin levels of RINm5F cells cultivated under FC and FD conditions. As anticipated, cells grown under FC condition for 1-week could synthesize substantial amounts of insulin as compared to FD control (2.3±0.1 v.s. 0.3±0.2 mU/L, respectively). In addition, we also challenged the cells grown in either FC and FD conditions with various concentrations of glucose in order to explore their responses on insulin secretion. Our results indicated that the secretion of insulin was apparently responsive to glucose stimulation in FC controls. However, this normal response of insulin secretion by glucose stimulation was lost when these cells were deprived of folate (Fig. 8B).

## Discussion

Despite a plethora of epidemiological reports revealing high prevalence of FD and hyperhomocysteinemia in patients with progressive type-2 diabetes [25], [26], there has been a paucity of evidence linking FD as an etiological factor for the early onset of diabetes. For this reason, we set out to investigate whether or not FD is a legitimate risk factor for the pathogenesis of diabetes. Using an insulin-producing RINm5F cells, a rat pancreatic islet cell type, as an experimental cell model, we present evidence here that FD is indeed a legitimate risk factor for the pathogenesis of diabetes. Cultivating RINm5F cells under FD condition for one to two-week, a large proportion of cells was found to undergo apoptosis. Concomitantly, these cells were shown to be no longer capable of synthesizing insulin. In addition, insulin secretion in response to the stimulation by glucose challenge was becoming blunt.

It has been reported that RINm5F islet cells were genetically endowed with extremely low expression of antioxidant enzymes, particularly in H_2_O_2_-metabolizing enzymes, such as CAT, GPx and peroxidoredoxin (Prdx) [11]–[13]. For this reason, it can be inferred that these cells should be comparatively more vulnerable to oxidative damages to mitochondria and endoplasmic reticulum (ER) owing to their severely compromised abilities to detoxify cell permeable H_2_O_2_. The consequence of this event could then trigger the unfolded protein response (UPR) leading to the occurrence of ER stress. We now demonstrated that folate-deprived RINm5F cells could trigger overproduction of NO mediated by NF-κB-dependent iNOS induction that could deplete ER Ca^2+^ leading to cytosolic Ca^2+^ overload (Fig. 4) and CHOP-mediated ER stress as in analogous to the literature reported elsewhere [27], [28]. Along this same vein, the downstream events of ER stress have been documented to evoke the downregulation of two prominent anti-apoptotic constituents including Bcl-2 and GSH [29]. Importantly, it has been suggested that Bcl-2 can modulate sensitivity to oxidative stress through the regulation of GSH content [30]. In agreement with these studies, we found that FD could also induce simultaneous downregulation of Bcl-2 and GSH depletion, a hallmark of ER stress response. Meanwhile, we also observed that Bax expression was angmented during an episode of FD. Thus, the Bax/Bcl-2 ratio was drastically increased in folate-deprived RINm5F cells that could foster apoptosis through caspase-3 activation (Fig. 5) leading to the occurrence of nucleosomal DNA fragmentation of 180–200 base pair multimer and a drastic increase in the TUNEL-positive cell population (Fig. 6).

Several reports have demonstrated that intracellular GSH depletion could result in mitochondrial dysfunction [31]–[33], with transmembrane potential breakdown followed by cytoplasmic Ca^2+^ deregulation. Furthermore, in some lung cancer cells, GSH depletion had been found to enhance Bax protein translocation to mitochondrial membrane and then sensitized cells to apoptosis [34]. In consistent with these reports, we demonstrated that FD condition could trigger severe depletion of intracellular GSH contents (>75%) after RINm5F cells were cultivated under FD condition for 2-week. Thus, FD-induced intracellular GSH depletion could similarly play a pivotal role in arbitrating apoptosis in RINm5F cells.

The potential benefits of intracellular GSH and its role against oxidative damage could be elaborated by the following facts. First, GSH, not synthesized in mitochondria, must be synthesized in the cytosol and transported into mitochondria by a specific transporter. Second, the oxidized GSSG is not retransported into the cytosol for its reduction to GSH. Third, CAT, a major enzyme for breakdown of H_2_O_2_, is absent in the mitochondria of mammalian cells. All of these facts underscore the importance of mitochondrial GSH against ROS-induced oxidative injury. Since the reserve of mitochondrial GSH is strictly regulated and with limited capacity, several ROS-dependent mechanisms may be capable of triggering GSH depletion. First, mitochondria are the major source of ROS in the cells. Superoxide (O_2_^−^) is generated by the operation of complex I and III in the matrix and is converted to H_2_O_2_ by MnSOD. In addition, FD could induce iNOS-mediated overproduction of NO. Under this situation, the depletion of GSH can then be proceeded via the interaction of NO and GSH:
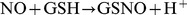



Alternatively, H_2_O_2_ can interact with the pre-existing ferrous (Fe^2+^) or copper (Cu^1+^) ions resulting in the formation of hydroxyl radical (OH^•^) via Fenton or Harber-Weiss reaction:
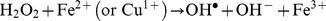


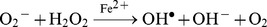



Hydroxyl radicals thus formed are capable of abstracting a hydrogen atom from GSH with commensurate formation of thiyl radical (GS•).
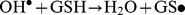



The depletion of GSH can then be proceeded via the termination reaction of GS• by colliding with another GS• resulting in the formation of GSSG, an oxidized form of GSH.
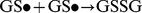



This potential pathway for GSH depletion engendered by FD in RINm5F cells was demonstrated to be feasible because deprivation of folate could robustically generate OH^•^ (Fig. 1D). Based on these arguments, we can conclude that FD-triggered GSH depletion may play a pivotal role in sensitizing RINm5F cells to a mitochondria- dependent apoptosis via the disruption of the mitochondrial potential (△Ψm) and increasing Bax/Bcl-2 ratio. GSH depletion resulting from oxidative stress that contribute to neuronal cell death and pathologic process has been demonstrated in neurogenerative disease including Parkinson’s disease (PD) and Alzheimer’s disease (AD) [35].

Insulin is a key hormone with an important role in the growth and development of tissues and the control of glucose homeostasis [36]. For this reason, it is utmost important to explore the possibility that FD condition can alter the ability of insulin- producing pancreatic islet RINm5F cells to synthesize this hormone. In this study, we have demonstrated that FD could impair the biosynthesis of insulin by RINm5F cells as reflected by the loss of insulin-FITC green fluorescence in confocal microscopic images and substantially decrease in insulin levels measured by immunoassay method (Figs. 7 and 8). More importantly, FD-triggered impediment of insulin biosynthesis was shown to be irreversible because replenishment of folate could not restitute the ability of these cells to resynthesize insulin. Along the same vein, FD could also blunt insulin secretion in response to the stimulation by glucose challenge (Fig. 8B).

**Figure 9:**
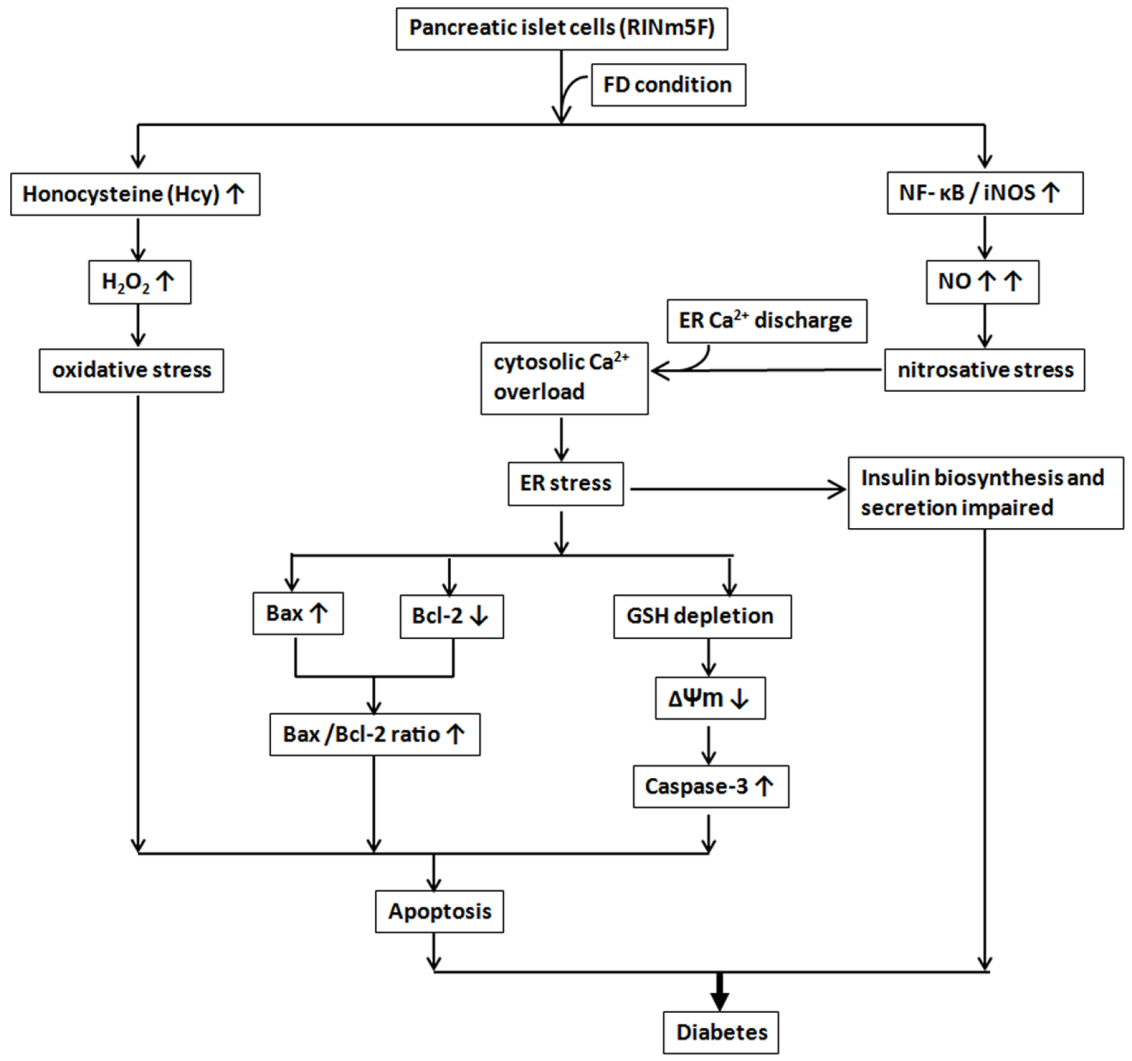
A diagrammatic representation depicting the involvement of diverse mechanistic pathways that arbitrating FD-triggered apoptosis of RINm5F cells.

In conclusion, we provide evidence here that FD condition could trigger oxidative-nitrosative stress in RINm5F cells through elevated ROS production due to Hcy accumulation and NF-κB-dependent iNOS-mediated NO production. The interplay of excessive NO and cytosolic Ca^2+^ deregulation subsequently triggered ER stress by activating CHOP expression. Concomitantly, ER stress provoked the downregulation of anti-apoptotic Bcl-2 in tandem with the upregulation of pro-apoptotic Bax expression and intracellular GSH depletion. These events consequently caused the disruption of mitochondrial membrane potential (△Ψm) and promoted the activation of caspase-3 leading to the final apoptosis of RINm5F cells. Even more importantly, we also uncovered that FD condition could severely impede the biosynthesis and the secretion of insulin. Taken together, our data provide strong evidence to lend support to the hypothesis that FD is a legitimate risk factor for the pathogenesis of diabetes. The involvement of diverse mechanistic pathways that orchestrating FD-triggered apoptosis of RINm5F cells are summarized in Fig. 9.
